# Feeling trapped and being torn: Physicians' narratives about ethical dilemmas in hemodialysis care that evoke a troubled conscience

**DOI:** 10.1186/1472-6939-12-8

**Published:** 2011-05-11

**Authors:** Catarina EC Fischer Grönlund, Vera Dahlqvist, Anna IS Söderberg

**Affiliations:** 1 Department of Nursing, Umeå University, 901 87 Umeå, Sweden; 2 Ersta Skondal University College, Department of Health Care Sciences P.O. Box 111 89, 100 61 Stockholm, Sweden

## Abstract

**Background:**

This study is part of a major study about difficulties in communicating ethical problems within and among professional groups working in hemodialysis care. Describing experiences of ethically difficult situations that induce a troubled conscience may raise consciousness about ethical problems and thereby open the way to further reflection.

The aim of this study was to illuminate the meanings of being in ethically difficult situations that led to the burden of a troubled conscience, as narrated by physicians working in dialysis care.

**Method:**

A phenomenological hermeneutic method was used to analyze the transcribed narrative interviews with five physicians who had varying lengths of experience in nephrology.

**Results:**

The analysis shows that physicians working in hemodialysis care suffered from a troubled conscience when they felt torn by conflicting demands and trapped in irresolution. They faced ethical dilemmas where they were forced to make crucial decisions about life or death, or to prioritize when squeezed between time restraints and professional and personal demands. In these ethical dilemmas the physicians avoided arousing conflicts, were afraid of using their authority, were burdened by moral responsibility and felt devalued and questioned about their way of handling the situation. The findings point to another way of encountering ethical dilemmas, being guided by their conscience. This mean sharing the agony of deciding how to act, being brave enough to bring up the crucial problem, feeling certain that better ways of acting have not been overlooked, being respected and confirmed regarding decisions made.

**Conclusion:**

The meanings of being in ethically difficult situations that led to the burden of a troubled conscience in those working in hemodialysis care, indicate the importance of increasing the level of communication within and among various professional groups - to transform being burdened by a troubled conscience into using conscience as a guide - in situations where no way of solving the problem seems to be good.

## Background

In the care of hemodialysis patients new ethical problems arise parallel with improved treatment methods for severely ill patients [1]. Uneasy feelings about giving uncomfortable orders and a lack of communication between physicians and other healthcare personnel, reported in ethical "rounds", were the point of departure for this study.

The head of a nephrological department in Sweden asked the Clinical Ethics Committee for help. One of the authors, (AS) who is the chair of the Committee, started to delineate a study in which physicians and nurses were asked to narrate situations of ethical difficulty that give rise to burdensome feelings of a troubled conscience. The theoretical framework for the study rests on the assumptions of Lindseth and Norberg [2] who claim that 'human beings live and act out of their morals, i.e. internalized norms, values and attitudes, without necessarily knowing about them'[2]. Internalized norms, values and attitudes may present themselves as "the voice of conscience" [3]. "Stress of conscience" may trigger positive changes but for some individuals a troubled conscience gives rise to a need to silence it which may lead to an increased risk of emotional exhaustion [4,5]. To give voice to what troubles one's conscience is one way of illuminating ethical difficulties that occur in a "high-tech" environment such as a hemodialysis ward. This study presents physicians' narratives concerning situations of ethical difficulty giving rise to burdensome feelings of a troubled conscience. The nurses' narratives will be presented elsewhere.

The development of hemodialysis as a way of prolonging life entails physicians in hemodialysis care facing new ethical problems [1]. Hemodialysis, hereinafter referred to as dialysis, has become available even for an agening population with end-stage renal disease [1] and the average age of Swedish patients in dialysis is now over 65 years [6].

For patients with end-stage renal disease, in a poor physical condition which minimises the chance of transplantation, dialysis has become a life-saving but regressive treatment with an extended course [7]. Cohen et al [8] think that dialysis is a both a life-prolonging therapy and a death-prolonging treatment [8]. Patients prescribed dialysis have to deal with several physical disabilities that affect their quality of life [1,9], such as ischemic heart disease, stroke [8] and peripheral vascular disease requiring amputation [10]. Being dependent on dialysis implies several changes in lifestyle and it is not uncommon for patients to become depressed and anxious about their wellbeing. A significant proportion of patients who need dialysis are unlikely to comply with the treatment [11]. To summarize, patients for whom dialysis will have problematic effects on their quality of life, generate ethical problems that their physicians have to deal with [1].

The high technology context of care is known to trigger ethical dilemmas concerning questions of life and death as treatments may cause more suffering if they prolongs the process of dying [1]. Several studies show that physicians experience ethical dilemmas concerning the withholding or withdrawing of life-sustaining treatments [12-14], especially in the case of patients with cognitive impairment that reduces their capacity to decide on their own good [12,13]. Withdrawal of treatment may be experienced as unethical as physicians have a responsibility and a duty to save life [14]. Holley et al [15] showed that physicians in nephrology in the United States feel they are insufficiently trained for end-of-life care. They feel inadequate with regard to psychological and existential aspects when caring for dying patients. Studies have reported physicians' assertions that guidelines would be helpful when making decisions about withdrawing or withhold treatment for incapacitated patients [12,13,16].

However, guidelines for withholding and withdrawing dialysis have already been published by the Renal Physicians Association and the American Society of Nephrology. The guidelines concern a process of decision making shared between physician and patient. This includes psychological considerations, planning, decisions and palliative care directed to achieving a good death [8,17]. Guidelines have also been developed in Sweden according to established ethical principles about withdrawing or withholding life-supporting treatment[18]. The purpose of the Swedish guidelines is to preserve respect for the patient's integrity and dignity and to determine whether or not life-support treatment would benefit the patient. They require a dialogue among the physicians, patient, relatives and healthcare personnel familiar with the patient. Ultimately the physician responsible for the patient has to decide according to her/his own judgment grounded in medical knowledge and reliable experience [18].

Despite these guidelines, physicians struggle with ethically difficult conflicts due to a lack of communication with healthcare personnel and relatives about decisions concerning the limitation of life-support treatment [19]. In a study by Oberle and Hughes [20] physicians described the difficulties of witnessing suffering and they felt uncertain about their course of action regarding patients and relatives. They felt burdened by having to make decisions and give uncomfortable orders. Söderberg [21] showed that when ethically difficult situations are not dealt with or when they are forced to act against their conscience healthcare personnel, experience severe frustration, later interpreted as having a troubled conscience.

Some ethicists state that conscience is a cornerstone of ethics [22], particularly in healthcare [3]. According to Glasberg [23] healthcare personnel often set high ideals for what they believe is good care. Being unable to carry out care according to their own ideals makes healthcare personnel question themselves and their morality [23]. In some situations, to avoid having a troubled conscience, healthcare personnel believe they have to break or bend laws and rules in their practice [24,25]. Several studies show that conscience is seen as an asset when one is able to express one's feelings, discuss them with others and act in accordance with one's conscience, but having to stifle one's conscience because of an inability to deal with moral problems was significantly related to burnout [21,23,24].

By telling stories involving ethically difficult situations evoking feelings of having a troubled conscience, it is possible to access the ethical thinking below the surface and bring it into the open for further consideration and reflection [2]. To date we have not found any study that describes situations involving ethical difficulties resulting in a troubled conscience among physicians in nephrology. Conscience points to the meaning of the ethical conflict which, when clarified, may be addressed in order to restore feelings of integrity and peace of mind [3].

The aim of the study, therefore, was to illuminate the meanings of being in ethically difficult situations that led to the burden of having a troubled conscience, as narrated by physicians working in dialysis care.

## Methods

### Methodological approach

When healthcare professionals experience ethically difficult situations, they are able to talk about them but are not usually able to spell out their morals. One way of investigating morals, according to Lindseth and Norberg [2] is to ask professionals to narrate situations involving regrettable conduct they have witnessed or participated in. To meet the aim of this study and be able to analyse the ethics expressed in stories the meanings of the experiences have to be revealed and studied. A qualitative approach was therefore deemed the most appropriate.

### Participants

The purposive sample comprised five physicians with 5 to 20 years (m = 10) experience in nephrological work employed at a hospital in Sweden. Some were specialists in nephrology and others were training to be specialists. The chief physician suggested other physicians based on variations in gender, age, length of work experience and experience of ethically difficult situations. The first author then met with these physicians as a group, informed them, verbally and in writing, about the study and their right to withdraw at any time without prejudice. The five physicians who agreed to participate were assured that their confidentiality would be protected. Approval to carry out the study was granted by the Ethics Committee of the Faculty of Medicine at Umeå University (03-499).

### Data collections

#### Interviews

Tape-recorded narrative interviews with each individual participant were performed in a private room at the nephrology department. Participants were asked to narrate any ethically difficult care situation that had affected their conscience. The open-ended interviews lasted 25-40 minutes without interruption and included follow-up questions, such as 'How did you feel then?" The audiotapes were transcribed verbatim, in Swedish, including pauses, laughs and sighs.

### Data analysis

#### Interpretations

The text was analyzed and interpreted using a phenomenological hermeneutic approach inspired by Paul Ricoeur [26], developed and described by Lindseth and Norberg [2]. The approach is useful when attempting to illuminate the meaning of lived experience through the interpretation of personal narratives. According to Ricoeur [26], a person's lived experience remains private but the meaning of it may be grasped through the interpretation of a narrative dialogue.

This analytical process comprises three phases, a naïve reading, a structural analysis and a comprehensive understanding, constituting dialectic movements between the whole and parts of the text, understanding and explanation and interpretation from what the text says to what it actually talks about [26]. First, a naïve reading was made which involved reading the text several times with an open attitude to arrive at a guess at the meaning of the text as a whole. The naïve reading indicated the direction the structural analysis should take. According to Ricoeur [26] in the naïve reading the researcher moves from the natural approach into a phenomenological approach which makes the researcher reflect on meaning. Second, in a structural analysis, the text was divided into meaning units. A meaning unit is a piece of the text of any length that conveys just one meaning. The meaning units were reflected on against the background of the naïve understanding, then condensed, reflected on, sorted according to similarities and abstracted into subthemes and themes. The themes were reflected on in relation to the naïve understanding to ascertain whether or not they validate or invalidate the naïve understanding. Third, a critical reading leading to a comprehensive understanding was formulated. This was based on the researchers' pre-understanding, naïve understanding, themes and sub-themes and was reflected on in relation to relevant literature. The authors first appropriated and interpreted the text individually, then discussed the interpretations made until agreement on the most credible interpretation was arrived at [27]. According to Ricoeur [26] the text is open to a variety of interpretations, one therefore has to choose the interpretations one can argue for. In the structural analysis the text was de-contextualized from the meaning. The naïve and comprehensive understandings were interpreted within the text's context [26].

## Results

### Naïve understanding

The narratives about ethically difficult situations that caused the physicians to feel burdened by a troubled conscience concerned situations when they felt hesitant and uncertain about their professional responsibility. When making crucial decisions about withholding or withdrawing dialysis, the physicians were trying to cope with ethical dilemmas. They found themselves torn by conflicting demands, felt indecisive, alone, unconfirmed and accused. The way they handled ethical dilemmas evoked a troubled conscience that made them feel they had failed their patients and the patients' relatives.

### Structural Analysis

Two themes and five sub-themes emerged from the structural analysis, describing ethical dilemmas in the nephrologists' daily work that gave rise to a troubled conscience. The dilemmas concerned crucial decisions about life and death regarding medically fragile and often cognitively impaired persons. The incompatibility of demands from patients, their relatives, co-workers and family caused tension. See table 1.

**Table 1:** Themes and sub themes

Subthemes	Themes
Avoiding arousing conflicts	Feeling trapped in irresolution
Being afraid of using one's authority	
Feeling the burden of sole responsibility	
	
Feeling squeezed between time restraints and professional and personal demands	Being torn by conflicting demands
Feeling doubted and unconfirmed by registered nurses (RNs)	

### Feeling trapped in irresolution

#### Avoiding arousing conflicts

The physicians had to make crucial decisions about life-support treatment when the patient and relatives were in disagreement. The narratives told of situations when a patient decided not to start or not to continue dialysis, but lacked the strength to resist an overbearing relative. The relative claimed to have the right to make the decision for the patient about whether dialysis treatment should start or continue. The physicians risked being caught in a conflict between the patient and the relative, and believed a conflict would threaten the patient's wellbeing. If the relative insisted on treatment the physicians did not want to intervene in a conflict and so had difficulty in upholding the patient's best interest. Physicians knew from experience that, once started, dialysis treatment could continue for a long time with the risk that the patient would become totally confused. Yet, in order to avoid a conflict, physicians hesitated to make the final decision to withdraw treatment. They felt trapped by demands from the patient, the patient's relatives and also from frustrated RNs who hear complaints from the patient.

'He started to get sores on his legs that didn't heal, which became more and more painful. We let the relatives know that he wanted to stop the treatment but they ignored him..... a daughter...was overbearing.....He suffered from an infection which he couldn't cope with and we were obliged to amputate one of his legs. The wound from amputation never healed and it became necrotic with an open wound area where the bone was visible. Despite that and repeated discussions she [the daughter] could not accept that there was nothing to be gained'

Another physician narrated a very similar story:

'I think one should have withdrawn all treatment because it became a long history, about a month or more before he died..... In retrospect, from an ethical perspective, I think we should have defended the patient, disregarded the daughter and taken the risk of being reported, but she was strong and overbearing, so we did as she wished. I still wonder if we did the right thing and who we did it all for, was it for her [the daughter's] sake or for his [the patient]? And I think we failed the old man [patient]'

When they reflected, physicians questioned the purpose of dialysis treatment and wondered who, after all, benefited from it. Their conscience was troubled which made them realize they had not been sensitive enough to the patient's wishes. Their intention was to do good by complying with the relatives' demands and avoiding conflicts. Instead, the patients suffered. The physicians wished they had been brave enough to help the patients and relatives understand what was best for the patient, even if it meant encountering conflict.

#### Being afraid of using one's authority

In these situations the physicians found that it was not medically defensible to start or continue dialysis, yet felt uncertain about their authority and the power of their words. They did not want to influence the patient's and relatives' choice and hesitated far too long before opening the question about withdrawing treatment for a fragile patient whose opinion was sometimes difficult to interpret due to dementia or ambiguous communication. According to the physicians, this was a life or death question; the patient's answer depended on how physicians raised the question

'We have some kind of power over life and death. Dying sometimes takes a while but it obviously creates frustrations and you always get the answers afterwards in some way... sometimes there can be something positive in such extended dying. Sometimes it can be painful'

The narratives told about patients, relatives and physicians from different perspectives and provided a variety of interpretations of the situation. The relative may have perceived some quality of life for the patient in her/his deteriorating condition and, therefore wanted dialysis to continue. From experience, the physicians could see the condition of the patient deteriorating, but maintained a defensive manner because they sympathised with the relatives and were afraid of influencing them too much. The physicians became uncertain, decided to continue dialysis and hoped that the relatives would gradually gain some insight.

The narratives speak of situations when the patient was very critically ill, suffering severely with no chance of improvement. The RNs complained because they had to be very attentive to the patients during dialysis and sometimes even had to restrain their arm to prevent them from hurting themselves. The physicians believed that patients and relatives were living with a kind of hope and did not want to force them to face reality, even though they wanted to suggest withdrawing treatment. They felt trapped in their uncertainty and afraid of using their authority.

'One woman started dialysis and as the year passed she suffered more and more from dementia. She and her husband had been living together for a long time and were quite isolated so the husband had to take on a great responsibility. Most of their life revolved around her and her care....we started a discussion about her life.... and I asked if there was really any motivation for keeping her alive on dialysis. In some way it felt as if we were giving her dialysis for his [the husband's] sake rather than for her own.... the nurses started to get frustrated because she was lying there without really understanding and sometimes they had to hold her arm straight to give her the treatment...He didn't want to withdraw treatment because he still felt she had a kind of quality of life when not on dialysis'

On reflection, physicians had troubled consciences because they realized they were afraid of exercising their authority to guide the patient and their relative in the most realistic direction. Physicians believed they did the right thing in giving the patient and relative enough time to let the best decision mature but, afterwards, it felt as though they had failed the patient.

#### Feeling the burden of sole responsibility

The physicians felt alone when having to make a critical decision. They experienced a lack of consensus. They tried to discuss things with each other but found it difficult because they had different values, professional experiences and opportunities to see alternatives. Thus, consensus was not easy to reach. Ultimately, the principal physician had to make the decision alone and take responsibility for the consequences, unsure if it was for the best. Less experienced physicians believed dialogue with colleagues with similar views would be helpful and wished they had more time for in-depth discussions in ethically difficult situations.

'*It is the person who makes the decision who has to be responsible. Therefore one should not be forced to make decisions that one cannot back'*

The narratives also concern the difficulties that can arise when the decision to withhold dialysis has been made but a physician with temporary responsibility begins dialysis without consulting the principal physician. That physician then has to shoulder the responsibility for a decision taken by another physician. Physicians know from experience, that once dialysis is started it is much more difficult to withdraw and a temporary treatment order may become permanent.

'If you are responsible for a long time, perhaps you can see a decision with different eyes from someone who is there temporarily and just walks in and sees the possibilities but may then walk out again'

On reflection, physicians may feel their own vulnerability, having to make decisions about life and death without support from their colleagues or superiors and to accept decisions made by someone else. Having to make crucial decisions alone leaves them with feelings of having failed the patient and a troubled conscience.

### Being torn by conflicting demands

#### Feeling squeezed between time restraints and professional and personal demands

The physicians felt inadequate because of lack of time and conflicts between ideals and reality. This concerned everyday situations describing a stressful work situation with high demands, an increasing administrative workload and reduced time with the patient. They wished there were enough time for careful discussions with patients and relatives in situations where crucial decisions had to be made. In the narratives, the physicians spoke about feeling squeezed in impossible situations and feeling inadequate when facing prioritizing patient care against necessary administrative tasks.

'The difficulty is what the aim is. What tasks do we have and how much time do we have? Sometimes it is not very reasonable. Quality controls are increasing, documentation will increase and paperwork takes more time. There is less time for patients so you really do not manage to do the work you should do. If you do not do everything, then you get a troubled conscience because if you do not manage to do everything then you do not feel quite easy.'

When reflecting on their work situation, physicians spoke about a troubled conscience brought on by feelings of inadequacy because of ambiguous goals. They experienced a moral duty to provide patients and their relatives with enough information, yet felt hindered by their administrative workload.

The physicians described demands to realise their own high expectations, tacit professional ideals of adequacy and great competence or skill. The narratives talked about feelings of inadequacy when they were unable to live up to their own high expectations and demands for competence, imposed by themselves or others. In order to achieve the necessary competence and search for medical information, physicians needed to use their personal time, which interfered with their private lives.

'You have high ambitions and cannot live up to them, due to external or personal reasons'

The narratives spoke of ambitions to engage in unfinished research and of being ashamed to ask already overloaded colleagues for help with unfinished work. The physicians felt a tacit demand not to burden colleagues with their unfinished work and struggled to complete tasks in their spare time. In this struggle, the physicians tried to find solutions by themselves instead of sharing their burden and asking for help.

'Feelings of insufficiency influence me completely; I work a lot in my spare time.'

The narratives spoke about demands and expectations from their family and from work causing feelings of being split between personal and professional demands. The experience of inadequacy emerged when they were involved in a conversation with a patient while being aware that their family was waiting for them or their children had to be collected from kindergarten. Being unable to give the patient enough time and defaulting on their own family because of lack of time created in these physicians a sense of being devalued.

' If you have children and have to collect them from kindergarten at five pm and you know that you do not have time to register the patients properly ...then you feel that you really are not handling your situation in life'

Reflecting on their work, the physicians realized that, although they tried to do their best to master all the situations, their conscience might still condemn them, pointing out that they should work harder and do better in order to live according to the tacit ideals of their profession.

#### Feeling doubted and unconfirmed by RNs

The physicians and RNs met the same patient, but in different situations and from varying perspectives. Often the physicians had seen them in the consulting room, sometimes for some years before the patients became dependent on dialysis. From experience, physicians knew that patients are usually healthier when they first encounter the physician, but that the situation may change. When dialysis is started patients are sometimes in a deteriorating state of health and often depressed. Physicians also know that during dialysis, the patient will usually entrust the RNs with their troubled life story but, a few days later, may tell the physician that everything is just fine.

'You see things from different angles. One thing is that patients behave strangely. They are here so many hours per week with the dialysis personnel and they meet the same nurse almost every time. The patients seem to be able to complain a lot to the RNs. Then when I, as a doctor, arrive ten minutes later, everything is going quite well for the patient. We get different information from the patient as well'

RNs' opinions about whether to start or continue dialysis treatment for a very critically ill patient was one area of dissension between RNs and the physicians. When the physicians decided to start or continue dialysis for a critically ill patient, they felt questioned and accused of failing the patient. They experienced a lack of respect or understanding from RNs, even if at times they felt uncertain about whether or not their decision was appropriate.

The narratives also revealed a major conflict between the curative and palliative aspects of dialysis. On the one hand, there is a curative view of dialysis which deems it a failure when a uremic person without transplantation options deteriorates and finally dies, often in a critical condition. On the other hand, dialysis may increase the patient's wellbeing resulting in improved appetite and increased energy, at least for some time. From a palliative perspective, dialysis treatment can be experienced as meaningful; however, there was a lack of consensus among the physicians or between the physicians and the RNs.

'We are after all working with palliative treatment and, of course, if that is one's approach, we start dialysis and the patients die anyway and it may be experienced as a failure but from another perspective it[treatment] may be meaningful. The patient and relatives have time to end their life'

Physicians perceived a distance between themselves and RNs and wished they could understand the RNs*' *intentions. They wanted to find a way to explain the reality of the patients*' *and relatives*' *situation. When obliged to defend their decisions, or decisions made by the physicians group with which they themselves may have disagreed, the physicians sometimes felt uncertain or ambivalent. Reflecting on these situations, their conscience was troubled because they had not stood up for their decisions.

## Discussion

The aim of the study was to illuminate the meanings of being in ethically difficult situations that led to the burden of having a troubled conscience, as narrated by physicians working in dialysis care. The findings show that the physicians felt *trapped in irresolution *when obliged to decide about withdrawing or withholding dialysis in the face of dissonant opinions. They experienced *being torn by conflicting demands *when ideals and reality clashed. The situations related in their narratives represented true ethical dilemmas in which physicians wanted to do good by avoiding doing wrong. In ethical dilemmas, however, there is no one truly good solution [3]. The physicians' choice was, therefore, not between doing good or bad but rather which would be the lesser of two evils [28]. When telling their stories physicians realized that by avoiding one evil they unintentionally opened the door to the worse evil by not being sensitive enough to the patient's wishes, failing the relatives by not bringing up the crucial problem for discussion and ultimately failing themselves by not being true to their own values. Ricoeur [28] says that in the concrete situation when conflicts between different demands clash and we not only have to choose between good or bad but rather between an evil and a lesser evil to protect life, it is important to validate one*'*s standpoint. Silfverberg [29] believes that an ethical dilemma makes us feel confused and uncertain because we do not know what to do, but we still feel bound to act with no rules to follow [29]. To find a clue as to what is best for the other in an ethical dilemma, it is essential to be sensitive to one's own attitude and clarify one's inner motives [29,30].

The physicians in this study tried to follow what they believed was a good way of handling the ethical dilemma caused by conflicting opinions. They tried to do good by avoiding conflict between patients and relatives and wanted to open the way for consensus while not influencing the relatives' opinions. In hesitating to make a final decision about withdrawal of treatment they hoped the patient and relative would arrive at the right decision. Instead of following their own conscience in giving the patient and relative guidance, the physicians said they kept out of the way. They felt they were hemmed in and, in avoiding taking action, assumed a defensive attitude. Lögstrup [30] claims that by continuously considering instead of acting in difficult situations we may escape uncomfortable moral obligations to take the initiative for change. It is easier to continue considering but to do it continuously robs us of the power to act. Nykänen [31] argues that if one's conscience knows what is right but one still does what one believes others expect, one is directed by a false conscience and ultimately turns against oneself. Disregarding one*'*s conscience means escaping from the true self and is often followed by feelings of guilt.

According to Fromm [32] when you are not sensitive enough to follow the voice of conscience, conscious feelings of guilt about the person being failed will be induced. Later on a whole complex of unconscious guilt feelings for failing oneself arises. In the midst of unconscious feelings of guilt the experience of being trapped is generated [32]. The presence of such feelings of unconscious guilt was traced in the interview situation when the physicians' expressed a desire for another way in which to meet an ethical dilemma. The physicians wished they had been more sensitive to their own conscience and had been brave enough to influence the relatives in order to avoid the patients' suffering. Ricoeur [28] claims that conscience comes both from outside and inside. Its function is to examine our actions with suspicion, the judgmental function of conscience, but also to give us attestation that we are a sufficiently ethical being, in other words our-power-to-be.

According to the authors' interpretation the physicians in this study wanted to be confirmed, by their conscience but also by their colleagues and RNs, when making decisions in ethical dilemmas. Lacking support from colleagues and understanding and respect from RNs, the physicians felt devalued. Sörlie [33] found that in ethical dilemmas in pediatric care physicians felt lonely and burdened by uncertainty and responsibility. As mentioned above facing an ethical dilemma means facing conflicting moral demands where no decision is totally good [3]. It means that we often need to consult not only our conscience but also others to ascertain that the decision is as good as it can be, given the circumstances. We need to feel assured that we have not overlooked better ways to act [4]. Analysing situations involving ethical dilemmas together with others opens the way for sensitivity to others' perspectives and promotes moral development [22]. Interviewing psychiatric care providers about having troubled a conscience Dahlqvist et al [34] found that being sensitive but having a realistic approach towards one's conscience enhanced reconciliation and an ability to feel "good enough".

In this study, physicians spoke about feelings of being burdened by having sole responsibility in situations involving decisions about life or death. When investigating the ways physicians dealt with challenges in their work Andrae [35] found that they are educated to master all situations, are generally expected to have answers to all questions [35] and to make medical decisions on their own [36]. Hansson [37] describes the medical profession as not having developed a collaborative culture with support and shared responsibility for patients. The physicians in this study did not only have difficulties in reaching a consensus with colleagues, they also described feelings of being questioned and blamed by RNs. Sörlie [38] showed that support, encouragement and shared feelings of uncertainty helped physicians to develop an insight and acceptance that in an ethical dilemma one has to deal with insoluble problems. A prerequisite for being able to endure sole responsibility was being able to share the agony of being morally responsible when things go wrong. Silfverberg [29] emphasizes that an ethical mind with a feeling for concerns and judgements can be developed not only through being sensitive to the voice of conscience but also by observing and being corrected, in a sense of togetherness, by other people. In such an ethical climate personal character and virtues may develop.

In this study some of the physicians discussed ethical dilemmas with colleagues whom they knew from earlier experience had similar opinions and with whom they would probably be able to reach a consensus. The physicians and RNs had various experiences and perspectives concerning the patient's situation which they seemed to have difficulties communicating about. That these professional perspectives were not shared became an obstacle to reaching a common understanding. Lindseth, et al [39] showed that physicians and RNs in Norway had differing ethical perspectives in relation to the patient but deeper reflection revealed that they had similar core values. When different perspectives can be seen as complementing each other, in-depth dialogue between and among various professionals allowed mutual understanding and ultimately consensus concerning acceptable actions [40]. Studying ethically difficult situations in intensive care, Söderberg [21] showed that an ethical dilemma can only have a good outcome in an atmosphere of consensus. Physicians who succeeded in implementing very difficult decisions shared the following characteristics; they dared to remain in difficult situations, acted respectfully towards their opponents, were open to criticism, created a feeling of solidarity and succeeded in discussing the situation in such a way that they could achieve consensus.

The number of participants in this study is small, only five physicians were asked for interviews. The reason for this is that seven RNs were also interviewed for the study. The extent and richness of the resulting interview text and findings, however, led us to divide the reporting of the study into two manuscripts. The results of the RNs' interviews and the comparison between the groups will, therefore, be reported elsewhere. Despite the small sample the findings make an important contribution to developing a way of encountering ethical dilemmas.

Ricoeur [26] claims that the methodological steps help to create a distance between the researcher and her/his pre-understanding. Such a distance cannot be realized completely [27,41] but becoming more aware of the situation through reflection helps to limit the bias [2]. The authors are all RNs working in the following fields: clinical ethical support for all groups of professionals in a County Council (AS); researching matters of conscience in healthcare (VD); and working in anaesthetic care for many years (CFG). All three authors were involved in the analytical process and focused attention on awareness of their own values in order to increase the credibility of the analysis [2].

Interviewing another professional may constitute a methodological limitation. However, there may also be a positive effect in that physicians might be more open and willing to explain more explicitly what they mean to another professional. Another professional may also be sensitive to aspects of the phenomenon that are taken for granted within one's own profession.

A further limitation is that the interviewees were preselected by the chief physician according to the criteria for inclusion. However, the chief physician asked the clinical ethics committee for help and he knew which of the physicians on the ward would meet the inclusion criteria and could provide rich narratives. The interviews were carried out at one of the few hospitals in northern Sweden where dialysis is performed, making it possible to identify the participants. In order to preserve their confidentiality, age and gender are excluded from the text. During the analysis process five sub-themes emerged which are linked. The sub-theme "Feeling squeezed between time restraints, professional and personal demands" covers conflicts concerning prioritization of time in everyday situations. It does not concern crucial decisions about life or death but crucial decisions about how to do good or be good.

The findings from this study cannot be generalised, but can probably be re-contextualized to other contexts where similar ethical dilemmas occur concerning the withdrawing or withholding of treatment, e.g. in intensive, oncology and emergency care. The findings can also be re-contextualized/transferred to other contexts where professionals have to live and deal with conflicting demands.

## Conclusion

In this study the physicians in dialysis care narrated situations where ethical dilemmas occurred and pointed to possible ways in which conscience could be used as a guide instead of being a burden. In facing ethical dilemmas these physicians suffered from a troubled conscience when they were torn by conflicting demands and trapped in irresolution, despite these feelings being a natural response to ethical dilemmas. This is because in ethical dilemmas there are no rules governing the actions to be taken, only an ethical demand to act [29].

In the ethical dilemmas narrated, the physicians were not only burdened by a troubled conscience, but were also challenged by feelings of being left alone, burdened with moral responsibility, not understood and questioned about their way of handling the dilemma. The physicians felt devalued when they were not confirmed by colleagues, RNs and their own conscience. The findings, however, point to another way of encountering ethical dilemmas - being guided by their conscience. This means sharing the agony of deciding how to act in ethical dilemmas; being brave enough to bring up the crucial problem among those involved, feeling certain that better ways to act have not been overlooked and being respected and confirmed regarding the decisions made. This study points to the importance of increasing the level of communication within and among varying professional groups when ethical dilemmas occur and no way of solving the problem seems to be acceptable. Further research is needed into how to communicate those overlooked values that the voice of conscience seems to draw our attention to.

## Competing interests

The authors declare that they have no competing interests

## Authors' contributions

CFG carried out the interviews, participated in the analysis and completed the manuscript VD read the interviews, participated in the analysis, helped to draft and complete the manuscript. AS designed the study, read the interviews, participated in the analysis, helped to draft and complete the manuscript. All authors have read and approved the final manuscript

## Pre-publication history

The pre-publication history for this paper can be accessed here:

http://www.biomedcentral.com/1472-6939/12/8/prepub
